# Clinicopathologic characteristics and outcome of childhood and adolescent Ewing’s sarcoma in center of Iran

**Published:** 2014-07-20

**Authors:** A Akhavan, F Binesh, A Hashemi, H Shamshiri

**Affiliations:** 1 Department Of Radiotherapy,Isfahan University Of Medical Sciences,Isfahan,Iran; 2 Department Of Pathology,Shahid Sadoughi University Of Medical Sciences,Yazd,Iran; 3 Department Of Pediatric Medicine, Shahid Sadoughi University Of Medical Sciences,Yazd,Iran; 4 General Practitioner, Shahid Sadoughi University Of Medical Sciences, Yazd, Iran

**Keywords:** Ewing’s sarcoma, Survival, Child, Iran

## Abstract

**Background:**

Ewing’s sarcoma family is a group of small round cells tumors. The aim of this study is to evaluate clinicopathologic characteristics and outcome of Ewing’s sarcoma in children and adolescents in Yazd, Iran.

**Materials and Methods:**

All patients under 19 years with documented pathology of Ewing’s sarcoma family tumor who referred to Shahid Ramazanzadeh Radiotherapy center between 2002 to 2010 were enrolled in this retrospective study. Overall survival and disease free survival and prognostic factors were evaluated.

**Results:**

Among approximately 80,000 patients who referred to Shahid Sadoughi pathology department, over an 8-year period, the total number of patients with Ewing sarcoma was 32, of which, 18 cases were under the age 19 . The mean age was 13.72 years. Five patients (27.8%) had metastatic disease at the time of diagnosis. Complete response had been achieved in 8 (44.4%) of the patients. Local recurrence occurred in 4 (22.2%) of the patients. During the follow up 13 (72.2%) of the patients showed metastases. The mean overall survival was 34.79 months (95% CI: 22.27-47.32) .One, two, four and five year survival was 72%, 39%, 25% and 17% respectively. Complete remission occurred in 10 patients (63.6%). A trend of better overall survival was found in these patients (p=0. 55). When the brain and bone metastases occurred, the overall survival decreased significantly (p=0. 003 ).

**Conclusions:**

The overall survival rate of Ewing's sarcoma is very low in comparison with other parts of the world.

## Introduction

Ewing’s sarcoma family tumors (ESFT) are a group of small round cell tumors that may arise from bones or soft tissue (1,2 ,3). Ewing’s sarcoma is the second most common tumor of bone in childhood and adolescence (1,2,3,4). The annual incidence of Ewing’s sarcoma is approximately 2 and 2.93 per million in theUK (5) and the US (6) respectively. The peak age of incidence of the disease is 15 years (7).About 80% of affected patients are under 18 years old (7) .Survival of Ewing’s sarcoma patients’ has increased since 1970 and at present, 5 year overall survival rate of localized disease is approximately 75% (6). There are few papers about Ewing’s sarcoma in Iran. As our knowledge, there are only two studies about Ewing’s sarcoma (8 ,9 ), however none of them reported survival rate. In this research we evaluated 18 children and adolescents with Ewing's sarcoma (the age range: 8 to18 years) and reported their clinicopathologic characteristics, progression free survival and overall survival.

## Materials and Methods

This study was approved by the Ethics Committee of Shahid Sadoughi University of Medical Sciences. All patients with documented pathology of Ewing’s sarcoma /primitive neuroectodermal tumor, under the age 19 years, who referred to Ramazanzadeh radiation oncology center, were enrolled in this study. Patients who were referred for treatment of relapsed disease were excluded. The study was begun in 2002 and ended in 2010. Surgery had been performed for some of the patients and chemotherapy had been started before referral for all of them. Chemotherapy had been continued during and after radiation therapy. Since chemotherapy of some of the patients had been performed in other centers such as private offices, we did not know the exact chemotherapy dosage of drugs and dose intensity; however, according to the patients’ hospital discharge notes and telephone contacts with their physicians, we found that all of the patients had received different combinations of Doxorubicin, Ifosfamide, Etoposide, Cyclophosphamide, Actinomycin and vincristine. Radiation therapy was performed by using Cobalt 60 machine or 9MV photon of Nepton linear accelerator. Treatment volume was defined according to prechemotherapy and /or preoperative imaging if accessible; otherwise it was defined according to scar of surgery and surgeons’ notes. For all patients 3 to 5 cm longitudinal and 2cm lateral margin added to the clinical target volume. At least a 5000cGY radiation dose was delivered. Variables recorded were the hospital patient registration number, date, name, age, sex, address, topography, resection margins, symptoms, some laboratory findings such as ESR and CRP, presence or absence of metastasis, treatment protocol ,overall survival, and time for recurrence for each patient according to the clinical data provided in their medical charts and patients follow up via phone. In this study the overall survival was defined from the day that pathological examination had been performed until death or last visit (or telephone call) and disease free survival from the day of ending all treatments to the first pathological, radiological or clinical evidences of relapse. 

**Statistical Analysis**

Statistical analysis was performed with SPSS 17.0 (SPSS Inc., Chicago, IL). Survival was estimated using the Kaplan-Meier method. Univariate and multivariate logistic regression analysis was used to determine any correlation between patient-related factors and outcome.

## Results

**Figure1:**
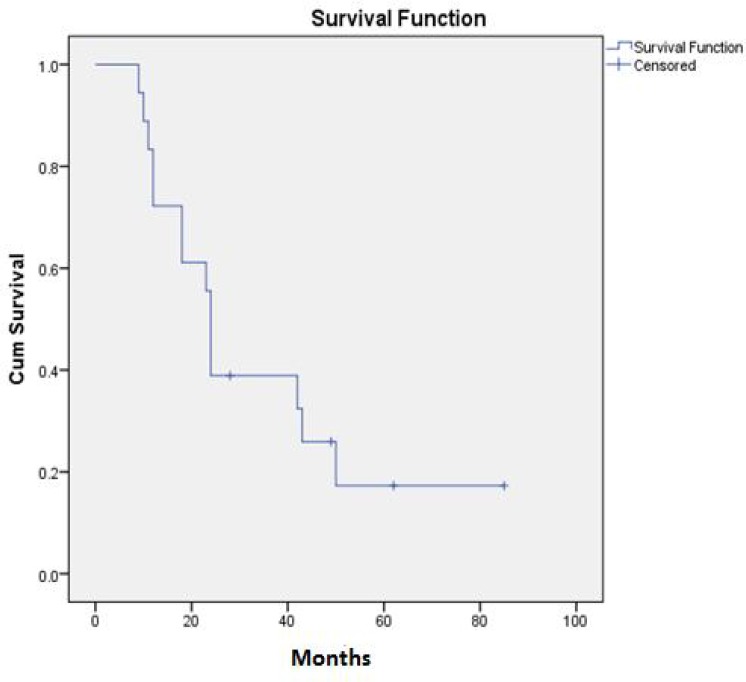
Overall survival of participants in the study

**Figure2:**
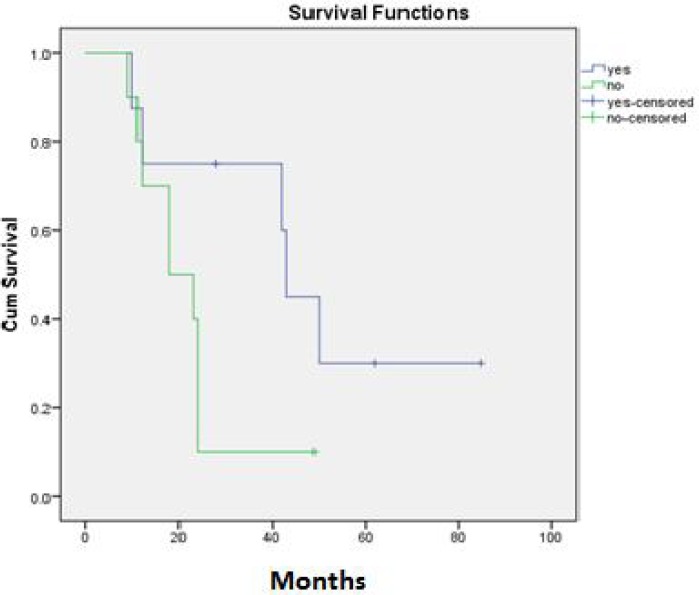
Association between complete remission and overall survival

**Figure 3:**
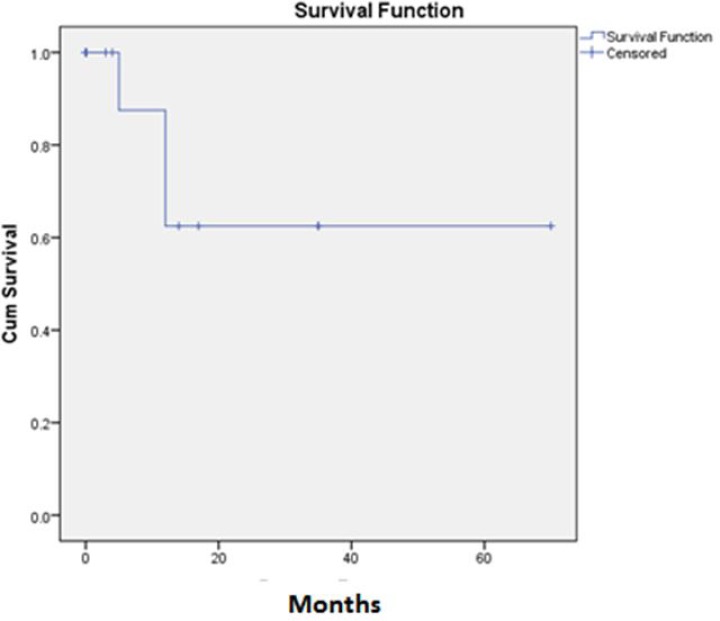
Disease Free survival

Of the 18 patients, 10 (55.6%) were girls and 8 (44.4%) were boys. The mean and median age was 13.72 and 15 years respectively. Mean age was 13.6 years (95% CI 10.79-16.41) and 13.88 years (95% CI 11.07-16.68) for girls and boys respectively. The site of disease was lower extremities in 44.4%, chest wall and lung in 22.2%, and pelvic, upper extremities and vertebras, each one in 11.1%. The most common symptoms were pain (83.3%) ,inflammation(33.3%) and claudication (11.1%) .Fifty present of the patients had abnormal ESR and 38.9% of them had positive CRP. Five patients (27.8%) had metastatic disease at presentation. Surgery had been performed in 14 (77.8%) of the cases and 11 of them had positive surgical margins. In 16 (88.9%) patients, radiation therapy for the primary site had been performed. Complete response (complete response means that signs of clinical or radiological disease resolved with chemotherapy or radiotherapy) had been achieved in 8 (44.4%) of the patients. Local recurrence occurred in 4 (22.2%) of the patients. During the follow up 13 (72.2%) of the patients showed metastases; bone metastases in 8 patients (44.4%) that three (16.7%) of them had brain metastases too, and lung metastases in 8 (44.4%) of the patients. In the summer of 2012 only 4 (22.2%) of the patients were alive. The mean overall survival was 34.79 months (95% CI: 22.27-47.32) and the median overall survival was 24 months. One, two and five year survival was 72%, 39% and 17%, respectively (fig-1). Among the variables, sex, primary site of the disease, primary symptoms at presentation, abnormal laboratory tests, presence of primary metastasis, surgical margin status and radiation therapy did not show a significant relationship to the overall survival. Complete remission (complete remission means that at the time of the study there were no signs of obvious clinical or radiological disease) occurred in 10 patients (63.6%) and there was a trend of better overall survival in these patients (p=055)(Fig-2).Therefore disease free survival (DFS) was computable for these ten patients. The mean DFS was 47.37 (95% CI: 27.08-67.66) and five year DFS was 61% (Fig-3). Occurrence of local relapse and lung metastases had no significant impact on the overall survival, however, in the presence of brain and bone metastases, the overall survival decreased significantly (p=0. 003 ).

## Discussion

In this study we investigated clinicopathologic characteristics and outcome of Ewing’s sarcoma in children and adolescents in Yazd, Iran. In accordance with some texts, in the presented study, the most common primary site of disease was lower extremities and the most common presentation symptom was pain (1). Our knowledge about Ewing’s sarcoma is very limited in Iran. As we found only one epidemiological study had been performed regarding childhood Ewing’s sarcoma in Khozestan. In this study 47 patients had been evaluated that most of them were between 10 to 15 years and the prevalence of some variables such as a primary site, local tenderness, palpable mass, pathologic fracture, fever, pain, limping, and weight loss had been investigated. Lung (13%), skeletal system (9%) and bone marrow (2%) were the most common metastatic sites (9). However, there was no report about survival rates and the variables that had impact on the survival. The mean overall survival in our study was 34.79 months (95% CI: 22.27-47.32) and the median overall survival was 24 months. One, two, four and five year survival were 72%, 39% ,25% and 17% respectively. Five year survival of European children and adolescence has increased during these three decades and has reached about 34% in 1978to 1982 to 66% in 1993 to 1997 (4) .According to Epidemiology and End Results (SEER) data in the US five-year survival improved from 36% for patients treated from 1973 to 1977 to 59% for the patients who treated from 1993 to 1997(1). A study was performed in Japan between 1981and 2003 and 243 Ewing’s sarcoma patients with the median age of 16 years were evaluated .In that study the 5 year overall survival and EFS were 48.7% and 40.7%, respectively (10). The patients under the age 16 years at presentation had an event free survival rate of 50.1% . In a single institution study in South Korea event free survival in the under the age 12 years group was 74.9% . This study had been carried out between 1986 and 2008 and the patients had a metastatic disease at presentation had been excluded from the study. Five year overall survival for the entire patient (less than 12 years or older) was 58.9%. (11)Another study had been performed in Central America between 2000 and 2009 on pediatric sarcoma. In that study, 175 cases suffered from Ewing’s sarcoma. Their 4 year overall survival was 36% (12).It is clear that outcome of our patients is the worst when we compare our results with these studies from the Europe, US, Asia and even Central America. Except Korean study, all of the other studies included the patients presented with metastatic disease. Overall metastatic disease at presentation is about 25% in the US (2) , 16.9% in Japanese’s study and 39% in Central America study. This item is 27.8% in our study. Since adding the chemotherapy to local treatment was begun in the 1970s, improvement of outcome is attributed to it. When new drugs such as Ifosfamide and Etoposide added to older chemotherapy regimen such as Doxorubicin, Vincristine and Cyclophosphamide, survival became better (13). In the late of 1970s and early 1980s two Intergroup Ewing Studies had been performed upon the patients presented with metastatic disease and they received Doxorubicin, Vincristine, cyclophosphamide , Actiomycin and radiation therapy with or without 5Flurouracil. Fiver year overall survival in both groups was about 30% (14).Although in these studies all of the patients were metastatic and new drugs such as Ifosfamide and Etoposide had not been used, the 5 year overall survival was better than our study. Ewing’s sarcoma is a chemotherapy and radiation therapy sensitive tumor, and surgery also had a role in local control of the large proportion of the patients, however the timing of local therapy is important. It is recommended that when the orthopedic /general surgeons are suspicious to Ewing’s sarcoma, a biopsy is taken, then the treatment begins with chemotherapy and after that local therapy (surgery +/- radiation therapy) is performed and finally chemotherapy will be continued for about 48 weeks. However, when surgery is carried out at the first step, there is possible that resection with a safe margin becomes impossible and chemotherapy is postponed until wound healing. This scenario has been happening for a significant proportion of our patients, and it is perhaps one of the reasons that in our study surgery could not improve the survival of the patients in contrast to other studies that show surgery increases the overall survival (10,15). On the other hand at least in non- metastatic disease, intensive chemotherapy improves the outcome (16). Since we are not aware about accuracy of prescribing chemotherapy in some of the patients, it maybe another reason of disappointing outcome. Finally our center was equipped with two dimensional radiation therapy system, and most of the patients referred to our center after surgery and some of them had inadequate preoperative imaging. These reasons could be cause of geographic miss, and at least one study showed inadequate target volume irradiation had an adverse effect on outcome (17). **Conclusion**

Our study showed that the overall survival rate of Ewing’s sarcoma patients in this province is very lower than other parts of the world and since it seems there is no significant difference between the natural of the disease in our region and the others, it is probably due to inadequate treatment. If a multidisciplinary team includes a pediatric oncologist, a radiation oncologist, an orthopedic surgeon, a pediatric a general surgeon, pathologist and a radiologist work together, probably the overall survival rate may increase. 

## Conflict of interest

The authors have no conflict of interest.
